# Biologics targeting IL-17 and IL-23 maintain stability in patients with psoriasis during COVID-19 infection: a case-control study

**DOI:** 10.3389/fmed.2023.1280965

**Published:** 2023-11-06

**Authors:** Dawei Huang, Yingyuan Yu, Jiajing Lu, Fei Tan, Yuling Shi

**Affiliations:** ^1^Shanghai Skin Disease Clinical College, Fifth Clinical Medical College, Anhui Medical University, Shanghai, China; ^2^Department of Dermatology, Shanghai Skin Disease Hospital, Tongji University, Shanghai, China; ^3^Institute of Psoriasis, Tongji University School of Medicine, Shanghai, China

**Keywords:** psoriasis, COVID-19, SARS-COV-2, biologics, disease management

## Abstract

**Background:**

Psoriasis is a chronic and refractory skin disease. The emergence of biologics provides more options for the treatment of psoriasis, but the COVID-19 pandemic poses challenges for the management of psoriasis.

**Objectives:**

The purpose of this study was to investigate the effect of different biologics on the stabilization of psoriasis during COVID-19 infection in China.

**Methods:**

This is a single-center, observational, retrospective, case–control study. Using our database, we conducted a remote dermatologic study by means of questionnaire follow-up or telephone follow-up to collect general information of patients, information related to COVID-19 infection and conditions of psoriasis for comparison and further analysis between groups.

**Results:**

Our study ultimately included 274 patients for analysis. We found that the patients in this collection had mild symptoms of COVID-19 infection, and only 13 of them needed to go to the hospital for medical treatment. Further studies found that in biologics, relative to tumor necrosis factor-α inhibitors (TNF-αi), interleukin-17 inhibitors (IL-17i) and interleukin-23 inhibitors (IL-23i) are both protective factors in flare-up of psoriasis [IL-17i: OR (95% CI) = 0.412 (0.189–0.901); IL-23i: OR (95% CI) = 0.291 (0.097–0.876)]. In addition, we also found that the proportion of people with increased psoriasis developing long COVID-19 increased, and we speculated that increased psoriasis may be a potential risk factor for long COVID-19.

**Conclusion:**

Our study showed that the use of IL-17i and IL-23i was a protective factor for psoriasis compared with TNF-αi, and could keep the psoriasis stable.

## 1. Introduction

Psoriasis is a chronic inflammatory disease. It is characterized by red papules or plaques covered with silver scales and is often accompanied by other health conditions (1). Psoriasis tends to recur and currently has no definitive cure. The pathogenesis of psoriasis is not yet fully understood, but there is a widespread belief that immune dysregulation, particularly dysfunction of the interleukin (IL)-23/IL-17 axis, plays a central role in its development (2). Additionally, epigenetics, specifically in terms of epidermal differentiation, has been found to be significant in psoriasis. Epigenetic mechanisms such as DNA methylation, histone modification, and the regulation of non-coding RNA also play a crucial role in the development of this condition (3). Psoriasis can be exacerbated by a variety of triggers, including infection (4). At present, the treatment of psoriasis has come to a new stage, and a large number of biological agents are applied in clinical practice, such as tumor necrosis factor-α inhibitors (TNF-αi, such as adalimumab, infliximab, etc.), IL-17 inhibitors (IL-17i, such as secukinumab, ixekizumab, etc.), IL-23 inhibitors (IL-23i, ustekinumab, guselkumab, etc.) (5).

The COVID-19 pandemic caused by SAR-CoV-2 presents significant challenges in managing psoriasis. Existing evidence suggests that patients with immune-mediated inflammatory diseases are more susceptible to contracting COVID-19 compared to the general population (6). Although there is currently no direct evidence linking psoriasis to an increased risk of COVID-19 infection or more severe disease outcomes, viral infections can potentially trigger the onset and exacerbation of psoriasis. Considering that COVID-19 infections result in an exaggerated immune response, this could disrupt the immune balance in psoriasis patients (7). Additionally, patients with moderate-to-severe psoriasis often undergo immunosuppressive therapy, which may negatively impact their susceptibility to developing COVID-19. However, recent studies have indicated that biologic agents used in psoriasis treatment, such as secukinumab, could potentially reduce the severity of COVID-19 infection (8). Despite the World Health Organization no longer considering COVID-19 a global health emergency, infections and outbreaks continue to occur. Nevertheless, it remains unknown how effectively biologic agents can manage psoriasis in the context of a COVID-19 infection.

Therefore, there is an urgent need to comprehend the impact of COVID-19 on patients who suffer from this prevalent, chronic immune-mediated skin disease. The aim of this study was to conduct a teledermatology investigation on the influence of the COVID-19 pandemic on patients with psoriasis, utilizing our database, in order to examine the impact of various biologics on the management of psoriasis during COVID-19 infection.

## 2. Methods

### 2.1. Study design

This is a single-center, observational, retrospective, case–control study. In this study, patients with psoriasis registered in the database of Shanghai Skin Disease Hospital from December 2022 to February 2023 were collected for telephone follow-up or questionnaire follow-up. Data on the severity and related symptoms of COVID-19 infection in patients with psoriasis, changes in psoriasis and treatment methods when infection were collected for retrospective analysis. This study was approved by the Ethics Committee of Shanghai Skin Disease Hospital, and all patients signed written informed consent when entering the database.

### 2.2. Patients

Included patients were at least 18 years of age with plaque psoriasis who were currently on long-term, stable biologic therapy. All patients were asked to undergo polymerase chain reaction (PCR) testing for SAR-COV-2, and positive patients were enrolled in the study. The duration of COVID-19 infection is defined as the time span from positive to negative PCR detection of the virus. Patients who were not treated with biologics during treatment and failed to complete a questionnaire or telephone survey will be excluded.

### 2.3. Study procedure

A retrospective analysis was conducted based on the data base of psoriasis patients and investigation results. Main information collection contents include: 1. Basic information of patients and types of psoriasis: name, gender, age and other demographic information; 2. We also collected information on the occurrence of COVID-19 infection, including symptoms of COVID-19 infection and corresponding treatment, given that the impact of COVID-19 infection on psoriasis remains uncertain and that some drugs have an impact on the condition of psoriasis (7, 9); and 3. The influence of psoriasis (flare-up or stable) and the use of biologics.

### 2.4. Statistical analysis

In light of the non-normal distribution observed in all numerical variables, we have chosen to represent them using the median along with the interquartile range (IQR). Conversely, for the classified variables, we have opted to represent them using the sample size (%) in our analysis. To compare between the two groups, we have employed the Wilcoxon test for numerical variables. On the other hand, if the variable was classified and the data satisfied the conditions of a theoretical frequency greater than 5 and a total sample size of 40 or more, we utilized the chi-square test for group comparison. Otherwise, we resorted to using Fisher’s exact test for group comparison. To identify statistically significant variables, a *p*-value of less than 0.05 was deemed significant, and these variables were then included in the multivariate logistic regression analysis. From this analysis, we calculated the odds ratio (OR) along with the 95% confidence intervals (95% CI). The significance level for all statistical tests conducted was set at 0.05. We conducted all statistical analyses using either SPSS version 26.0 or R version 4.2.1.

## 3. Results

**Figure 1:**
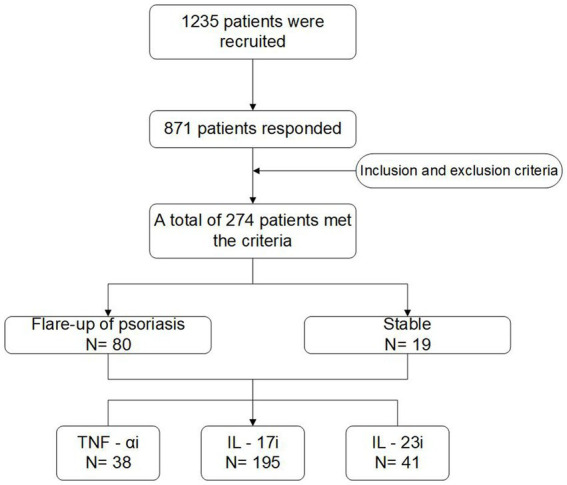
The flow diagram of this study. TNF-αi, tumor necrosis factor-α inhibitors; IL-17i, interleukin-17 inhibitors; IL-23i, interleukin-23 inhibitors.

The study flow chart is shown in Figure 1. A total of 1,235 patients were invited to participate in the study, a total of 871 patients responded, and 274 patients were included in the final descriptive analysis after screening based on inclusion/exclusion criteria.

### 3.1. Patient condition

Of the 274 patients, 80 had an exacerbation of psoriasis and 194 were stable (Table 1). Among the patients with an exacerbation of psoriasis, the use of IL-23i, IL-17 and TNF-αi were 10.0, 68.8% and 21.2%, respectively. Thus, a higher proportion of patients treated with IL-17i and IL-23i had stable disease than those treated with TNF-αi. Compared with the two groups, patients with aggravated psoriasis had a higher infection rate of COVID-19 in the past, and the difference was statistically significant (*p* < 0.05). There were no significant differences in other conditions, such as age, body mass index (BMI), gender, occupation, COVID-19 vaccine injection, past pulmonary disease and past allergic disease (*p* > 0.05).

**Table 1:** Comparison of condition in flare-up and stable psoriasis patients.

Characteristics	Stable (*n* = 194)	Flare-up (*n* = 80)	*p* value
Age, median (IQR)	43.5 (35.3, 58.0)	41.5 (34.0, 53.0)	0.196
Female, *n* (%)	55 (28.4%)	22 (27.5%)	0.887
BMI, median (IQR)	23.7 (20.9, 27.0)	23.4 (21.1, 27.7)	0.954
Occupation, *n* (%)			0.093
Outdoor	178 (91.8%)	68 (85.0%)	
Indoor	16 (8.2%)	12 (15.0%)	
Previous COVID-19 infection, *n* (%)	95 (49.0%)	52 (65.0%)	0.016
Covid-19 vaccinations, *n* (%)			0.759
3 shots	73 (37.6%)	26 (32.5%)	
2 shots	58 (29.9%)	28 (35%)	
1 shot	5 (2.6%)	3 (3.8%)	
Not shot	58 (29.9%)	23 (28.7%)	
Psoriasis treatments, *n* (%)			0.041
IL-23i	33 (17.0%)	8 (10.0%)	
IL-17i	140 (72.2%)	55 (68.8%)	
TNF-αi	21 (10.8%)	17 (21.2%)	
Lung disease, *n* (%)	61 (31.4%)	25 (31.2%)	0.975
Allergic disease, *n* (%)	75 (38.7%)	32 (40.0%)	0.836

*n*, number; BMI, body mass index; IQR, interquartile range; TNF-αi, tumor necrosis factor-α inhibitors; IL-17i, interleukin-17 inhibitors; IL-23i, interleukin-23 inhibitors.

### 3.2. Influence of COVID-19 infection on psoriasis

The patients we collected were generally not severely infected with COVID-19, with only 13 requiring hospital visits (Table 2). When COVID-19 was infected, patients showed a variety of symptoms. Compared with patients with stable psoriasis, patients with aggravated psoriasis were more likely to have fever, dizziness, weakness, muscle soreness, increased heart rate, dyspnea, chest tightness and chest pain, and the difference was statistically significant, and they have longer duration of COVID-19 infection (Table 2).

**Table 2:** Symptoms and severity of COVID-19 infection in different groups.

Characteristics	Stable (*n* = 194)	Flare-up (*n* = 80)	*p* value
Hospital visits, *n* (%)	7 (3.6%)	6 (7.5%)	0.287
Duration of COVID-19 (day), median (IQR)	7.0 (5.0, 8.0)	7.5 (7.0, 10.0)	0.008
Symptoms			
None, *n* (%)	8 (4.1%)	1 (1.2%)	0.401
Fever, *n* (%)	151 (77.8%)	72 (90.0%)	0.019
Maximum (°C), median (IQR)	38.6 (38.2, 39.0)	38.7 (38.2, 39.4)	0.370
Duration (hour), median (IQR)	36.0 (19.0, 48.0)	44.0 (12.0, 60.0)	0.870
Dizziness, *n* (%)	46 (23.7%)	33 (41.2%)	0.004
Headache, *n* (%)	60 (30.9%)	34 (42.5%)	0.067
Fatigue, *n* (%)	112 (57.7%)	57 (71.2%)	0.036
Muscle soreness, *n* (%)	98 (50.5%)	52 (65.0%)	0.029
Cough, *n* (%)	127 (65.5%)	58 (72.5%)	0.258
Coughing up phlegm, *n* (%)	80 (41.2%)	37 (46.2%)	0.446
Sore throat, *n* (%)	55 (28.4%)	30 (37.5%)	0.137
Catarrh, *n* (%)	65 (33.5%)	35 (43.8%)	0.109
Decreased oxygen saturation (≤93%), *n* (%)	0 (0.0%)	1 (1.2%)	0.292
Increased heart rate, *n* (%)	15 (7.7%)	15 (18.8%)	0.008
Shortness of breath (average > 30 beats/min), *n* (%)	1 (0.5%)	3 (3.8%)	0.140
Dyspnea, *n* (%)	2 (1.0%)	5 (6.2%)	0.039
Chest tightness, *n* (%)	8 (4.1%)	13 (16.2%)	< 0.001
Chest pain, *n* (%)	4 (2.1%)	8 (10.0%)	0.009
Abnormal sense of taste/smell, *n* (%)	40 (20.6%)	25 (31.2%)	0.060
Nausea and vomiting, *n* (%)	15 (7.7%)	8 (10.0%)	0.538
Diarrhea, *n* (%)	26 (13.4%)	14 (17.5%)	0.382
Secondary bacterial infections, *n* (%)	12 (6.2%)	8 (10.0%)	0.270
Others, *n* (%)	3 (1.5%)	1 (1.2%)	>0.999

*n*, number; IQR, interquartile range.

After COVID-19 infection, patients received drugs to improve their symptoms of infection through various means, and only 60 patients did not use any drugs. In addition, there was a difference between the two groups in the use of cough expectorants and antibiotics, and the use rate was higher in the group of patients with increased psoriasis (Table 3).

**Table 3:** Drugs used during COVID-19 infection in patients with psoriasis.

Characteristics	Stable (*n* = 194)	Flare-up (*n* = 80)	*p* value
None, *n* (%)	47 (24.2%)	13 (16.2%)	0.147
Proprietary Chinese medicine, *n* (%)	34 (17.5%)	18 (22.5%)	0.340
NSAIDs, *n* (%)	122 (62.9%)	58 (72.5%)	0.127
Cough and expectorant, *n* (%)	39 (20.1%)	25 (31.2%)	0.047
Antivirals, *n* (%)	0 (0.0%)	1 (1.2%)	0.292
Antibiotic, *n* (%)	12 (6.2%)	14 (17.5%)	0.004

*n*, number; NSAIDs, non-steroidal anti-inflammatory drugs.

**Figure 2:**
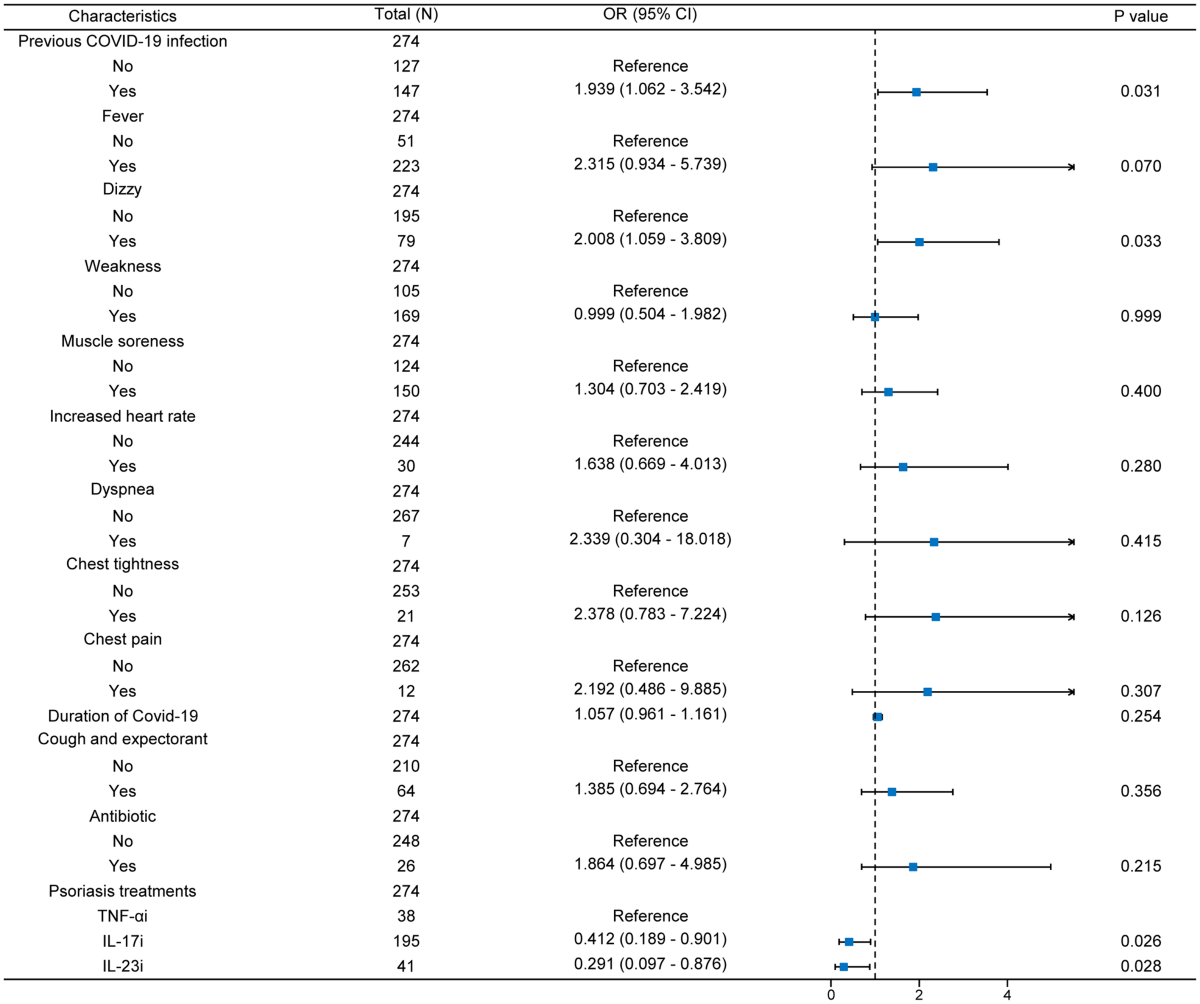
Multivariate logistic regression forest map. N, number; TNF-αi, tumor necrosis factor-α inhibitors; IL-17i, interleukin-17 inhibitors; IL-23i, interleukin-23 inhibitors; OR, odds ratio; 95% CI, 95% confidence intervals.

After this, we performed multivariate logistic regression for all variables with *p* < 0.05 (Figure 2). We found that after adjusting for confounding factors, patients with a history of previous COVID-19 infection and dizziness at the time of infection were more prone to exacerbation of psoriasis. It is worth noting that the use of IL-17i and IL-23i was a protective factor for psoriasis compared with TNF-αi, and these two biologics were able to stabilize psoriasis during COVID-19 infection [IL-17i: OR (95% CI) = 0.412 (0.189–0.901); IL-23i: OR (95% CI) = 0.291 (0.097–0.876)]. In addition, statistical analysis also showed that previous COVID-19 infection and dizziness during infection were contributing factors to the exacerbation of psoriasis (*p* < 0.05).

### 3.3. The long COVID

COVID-19 infection is not only a short-term impact, but also a long-term impact on patients even after PCR results turn negative. 202 of our patients continued to have a variety of symptoms after going negative. And it’s more common in people with advanced psoriasis, including persistent fever, slow thinking/poor concentration, fatigue, persistent cough, chest tightness, and insomnia (Table 4). Therefore, the condition of psoriasis may also affect the rate of long COVID.

**Table 4:** Long COVID in psoriasis patients with flare-up versus stable.

Characteristics	Stable (*n* = 194)	Flare-up (*n* = 80)	*p* value
None, *n* (%)	59 (30.4%)	13 (16.2%)	0.015
Persistent fever, *n* (%)	0 (0.0%)	5 (6.2%)	0.003
Dizziness, *n* (%)	14 (7.2%)	10 (12.5%)	0.160
Headache, *n* (%)	4 (2.1%)	6 (7.5%)	0.068
Slow thinking/poor concentration, *n* (%)	11 (5.7%)	12 (15.0%)	0.011
Fatigue, *n* (%)	65 (33.5%)	40 (50.0%)	0.011
Muscle soreness, *n* (%)	13 (6.7%)	9 (11.2%)	0.208
Persistent cough, *n* (%)	69 (35.6%)	40 (50.0%)	0.026
Coughing up phlegm, *n* (%)	47 (24.2%)	18 (22.5%)	0.760
Shortness of breath, *n* (%)	5 (2.6%)	1 (1.2%)	0.819
Chest tightness, *n* (%)	6 (3.1%)	10 (12.5%)	0.006
Chest pain, *n* (%)	1 (0.5%)	3 (3.8%)	0.140
Increased heart rate, *n* (%)	12 (6.2%)	9 (11.2%)	0.152
Nausea and vomiting, *n* (%)	4 (2.1%)	2 (2.5%)	>0.999
Diarrhea, *n* (%)	8 (4.1%)	2 (2.5%)	0.766
Abnormal sense of taste/smell, *n* (%)	27 (13.9%)	15 (18.8%)	0.313
Secondary bacterial infections, *n* (%)	6 (3.1%)	2 (2.5%)	>0.999
Insomnia, *n* (%)	3 (1.5%)	6 (7.5%)	0.032

*n*, number.

## 4. Discussion

The American Academy of Dermatology has outlined guidelines for the best management of psoriasis patients during the COVID-19 pandemic (10). It suggested that treatment for psoriasis and/or psoriatic arthritis does not appear to meaningfully alter the risk of acquiring a worse outcome from COVID-19 infection. Therefore, in most cases, patients who are not infected with COVID-19 should continue their biologic or oral therapy for psoriasis and/or psoriatic arthritis (10). However, the impact of COVID-19 infection on psoriasis remains uncertain, so it is critical for patients with chronic psoriasis to use a treatment to maintain stability during COVID-19 infection. There is actually very little data on the use of biologics to treat psoriasis patients during COVID-19 infection (11).

In this study, we found that the use of IL-17i and IL-23i was a protective factor for psoriasis compared with TNF-αi and was able to maintain stable psoriasis. Previous studies have demonstrated that IL-17i and IL-23i exhibit greater efficacy and lower risk of infection compared to TNF-αi (5, 12). Therefore, it is speculated that this could explain the enhanced disease stability observed in patients administered IL-17i and IL-23i during COVID-19 infection.

With the development of immunological research, biologics are used more and more frequently in the clinical diagnosis and treatment of psoriasis. Studies have shown that secukinumab can reduce the expression of angiotensin-converting enzyme 2 (ACE2) in the skin (the gateway of COVID-19 invasion into body), so it can be speculated that secukinumab can reduce the infection of COVID-19 (8). Previous studies have shown that people receiving IL-23i are less likely to develop infection and it does not affect the efficacy of SARS-COV-2 vaccine (13). And a review recommended IL-23i for the treatment of biologics during the COVID-19 pandemic (14). However, a cohort study showed that Patients treated with TNFi showed significant impaired serological response to SARS-COV-2 vaccine (15). Therefore, it can be speculated that IL-17i and IL-23i may reduce the influence of COVID-19 on psoriasis by reducing the influence of COVID-19 infection, so that the condition of psoriasis remains stable during infection, while TNF-αi, on the contrary, may lead to the exacerbation of the condition of psoriasis. This hypothesis is consistent with our findings. In addition, some studies illustrate that patients infected with COVID-19 exhibited elevated plasma levels of IL-23 and IL-17, along with increased levels of IL-17A-producing CD4^+^ and CD8^+^ T lymphocytes (16, 17). These findings indicate that targeting Th17 could potentially alleviate inflammation associated with severe COVID-19.

In addition, it was interesting to find that patients with increased psoriasis had a higher proportion of long COVID and varied manifestations, with long-term effects on patients’ lives. Since the causes and risk factors for long COVID are still debated, we speculate that the exacerbation of psoriasis in patients may be one of the risk factors for long COVID, although further research may be needed on this point. One thing is worth noting that patients who have had a long COVID still have higher plasma levels of IL-17, even after two years, even after they have passed the acute infection phase (18, 19). This suggests that the IL-17-mediated immune response may be associated with long COVID, and it may also indicate that patients have an enhanced IL-17-mediated immune response after psoriasis exacerbation, which may explain psoriasis flare-up as one of the risk factors for long COVID.

The study has several limitations. First of all, this study is a single-center study with a small sample size, and a larger sample size is needed to replicate our findings. Second, this study was retrospective, including selection bias associated with teledermatology investigations and patient reported results recall bias. Additionally, no other infections were recorded during the administration of biologics. Finally, we have a short collection time for symptoms after recovery from COVID-19, and longer follow-up may be needed in the future to identify the association between psoriasis and COVID-19.

## 5. Conclusion

Although the definitive cause of the association between COVID-19 infection and psoriasis is unknown, COVID-19 infection may be a trigger for the onset of psoriasis. Our study showed that the use of IL-17i and IL-23i was a protective factor for psoriasis compared with TNF-αi, and could keep the psoriasis stable. We therefore recommend the use of IL-17i and IL-23i for long-term treatment of psoriasis during COVID-19 infection. Moreover, patients with aggravated psoriasis had a higher proportion of Long COVID-19, suggesting that the impact of COVID-19 on patients with psoriasis may not be limited to the skin.

## Data availability statement

The original contributions presented in the study are included in the article/supplementary material, further inquiries can be directed to the corresponding authors.

## Ethics statement

The studies involving humans were approved by Ethics Committee of Shanghai Skin Disease Hospital. The studies were conducted in accordance with the local legislation and institutional requirements. The participants provided their written informed consent to participate in this study.

## Author contributions

DH: Writing – original draft. YY: Writing – original draft. JL: Writing – review & editing. FT: Methodology, Resources, Writing – review & editing. YS: Writing – review & editing.
